# Case Report: An unusual case of small bowel volvulus associated with pneumatosis intestinalis

**DOI:** 10.12688/f1000research.73042.1

**Published:** 2021-09-21

**Authors:** Alia Zouaghi, Dhafer Hadded, Mesbahi Meryam, Yazid Benzarti, Mona Cherif, Haithem Zaafouri, Khalil Ben Massoud, Chiraz Chamekhi, Anis Ben Maamer

**Affiliations:** 1 Department of General Surgery, Habib thameur Hospital, Tunis, Tunis, 1000, Tunisia; 2 Department of Radiology, Habib Thameur Hospital, Tunis, Tunis, 1000, Tunisia

**Keywords:** Pneumatosis cystoid intestinalis, small bowel volvulus, acute abdomen, case report

## Abstract

Pneumatosis cystoid intestinalis is a rare disease reported in the literature affecting 0.03% of the population. It has a variety of causes and its manifestation may change widely. It usually presents as a marginal finding resulting from various gastrointestinal pathologies. In the acute complicated form of pneumatosis intestinalis, management is challenging for physicians and surgeons.

We present a case of a 60-year-old patient who was admitted to our surgical department with a symptomatology suggestive of small bowel occlusion. Computed tomography demonstrated ileal volvulus associated with parietal signs suffering and pneumoperitoneum. An emergent exploratory laparoscopy followed by conversion was performed demonstrating segmental ileal pneumatosis intestinalis secondary to a small bowel volvulus due to an inflammatory appendix wrapping around the distal ileum. Further, detorsion, retrograde draining, and appendectomy were performed because there were no signs of necrosis and the appendix was pathological. The postoperative course was uneventful.

This case is exceedingly rare in the literature, because it was featured by the ileal volvulus due to appendicitis.This case report emphasizes the importance of surgical procedures in the management of symptomatic pneumatosis intestinalis.

## Introduction

Pneumatosis cystoid intestinalis (PCI) is a low-incidence pathology defined by the existence of air in the small intestine or colon wall. ^1^ PCI can affect any portion of the gastrointestinal tract and could be present in any layer such as the mucosa, submucosa, or subserosa. ^1– 3^ It can either be presented as a secondary form in 85% of cases or an idiopathic form in 15% of cases. ^1, 2^ The secondary pattern occurs more frequently in gastrointestinal causes such as bowel obstruction. ^3, 4^ The management of PCI is challenging to surgeons especially in symptomatic cases. ^5^ We report a rare case of ileal pneumatosis cystoides associated with small bowel volvulus, presenting with acute abdominal pain. This case is exceedingly rare in the literature, because it was featured by the ileal volvulus due to appendicitis.

## Case report

**Figure 1.**
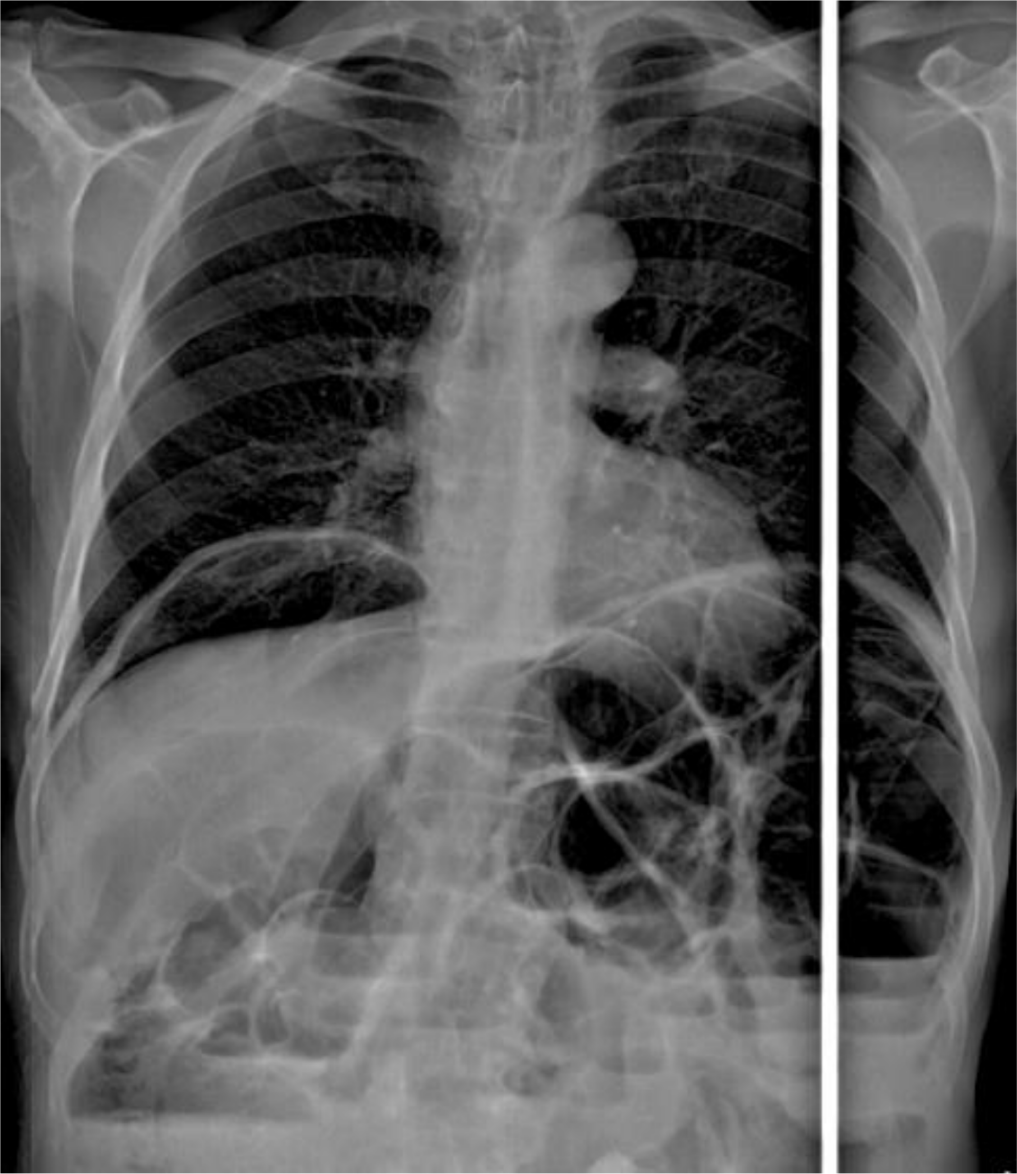
Dilated small bowel, multiple fluid levels and pneumoperitoneum on X-ray.

A 60-year-old retired, north African male patient without any medical or surgical history consulted the emergency department for 24 hours of abdominal pain, distension, and vomiting. The patient had experienced this pain a year earlier, but did not consult any doctor, and the pain faded away spontaneously. On physical examination, tachycardia and distended abdomen with mild tenderness were noted. White blood count was 8840 E/mm ^3^ and C reactive protein was 36 mg/l (normal values: White blood count: 4000E/mm ^3^, and C reactive protein: 1 mg/l). X-ray of thorax and abdomen showed dilated small bowel, multiple fluid levels and pneumoperitoneum ( Figure 1).

**Figure 2.**
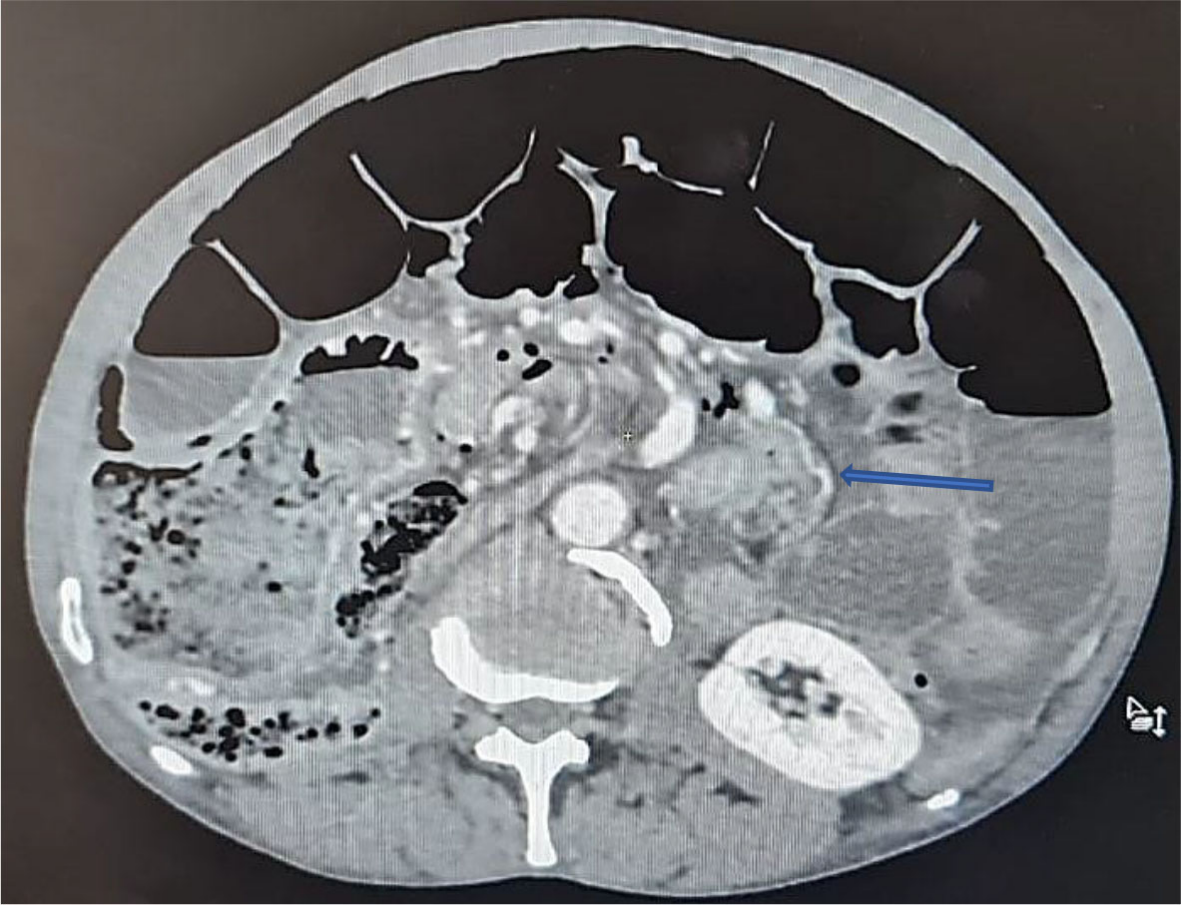
Ileal volvulus: dilated bowel segments associated with whirl sign.

**Figure 3.**
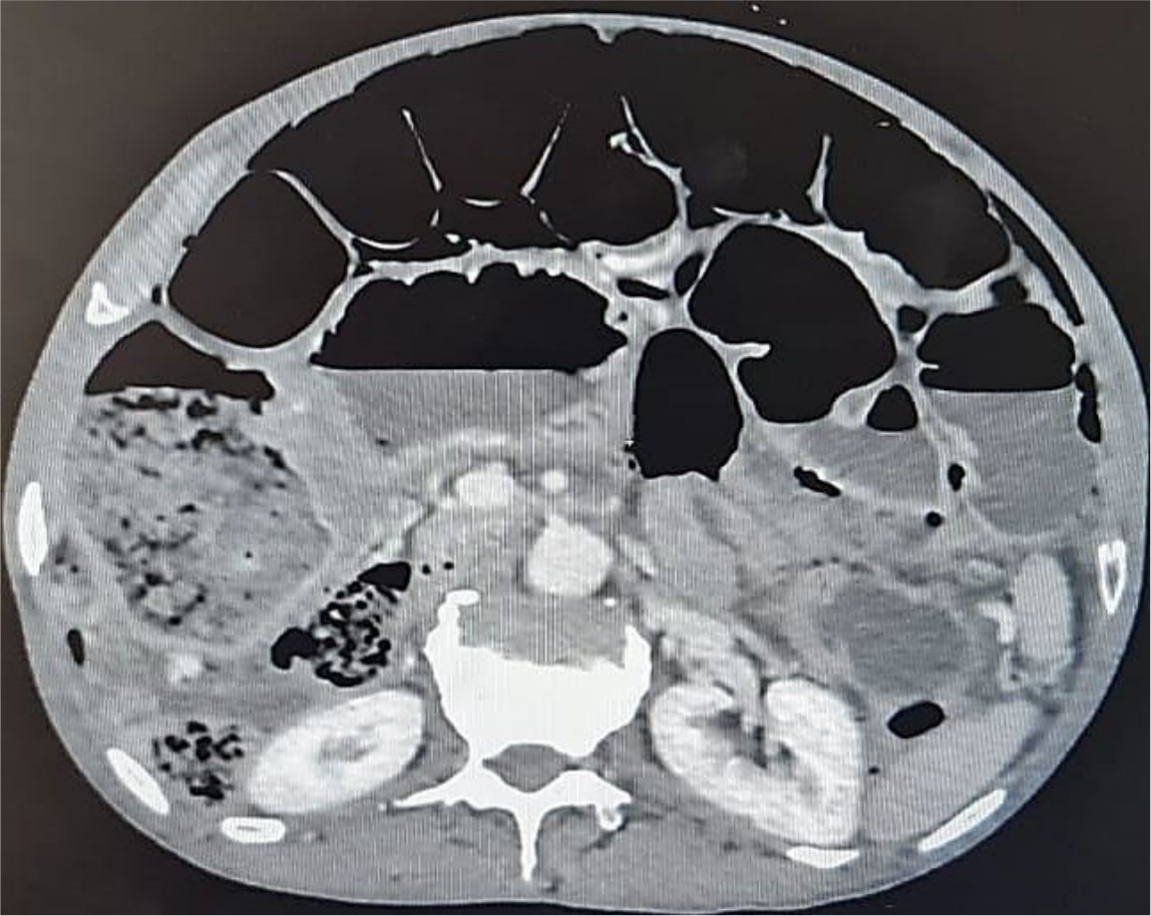
Multiple bubble lesions among ileal loops on Abdominal CT.

**Figure 4.**
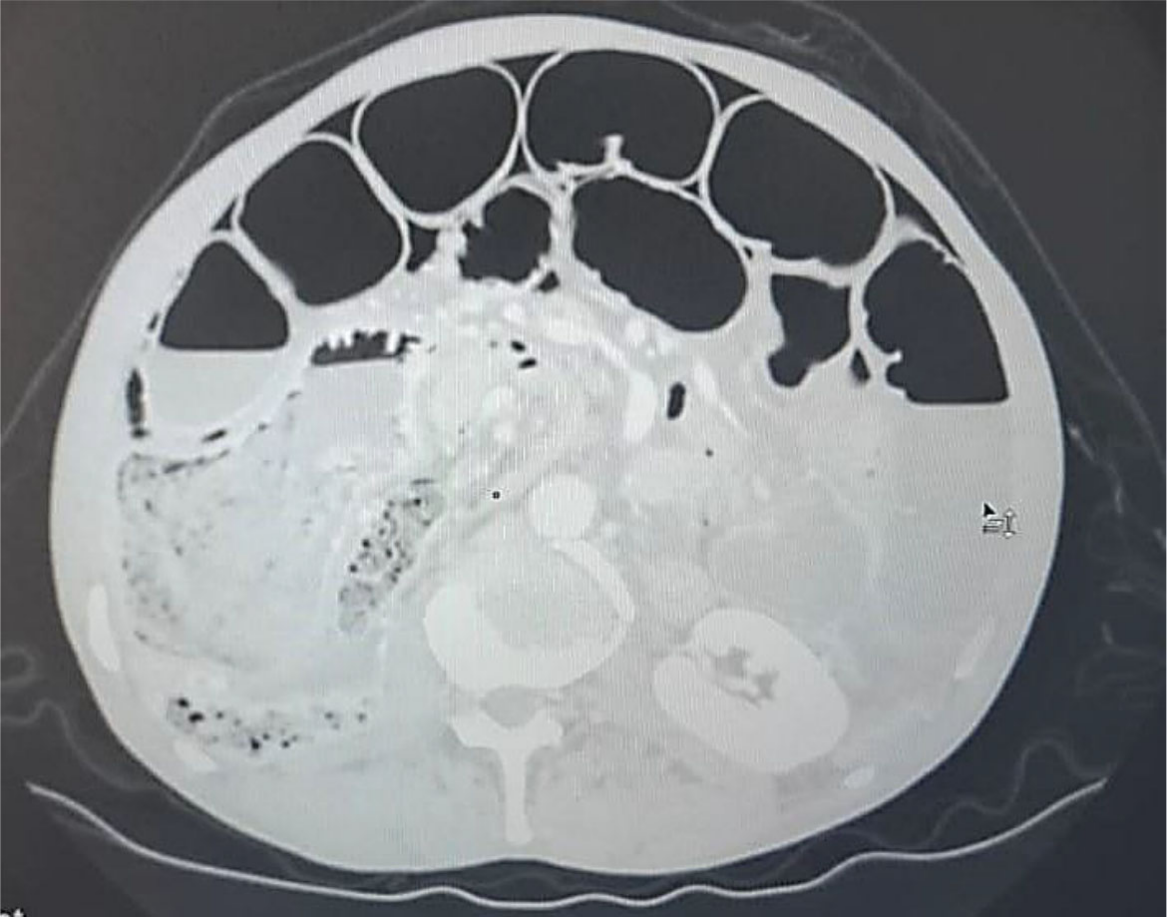
Abundant pneumoperitoneum.

**Figure 5.**
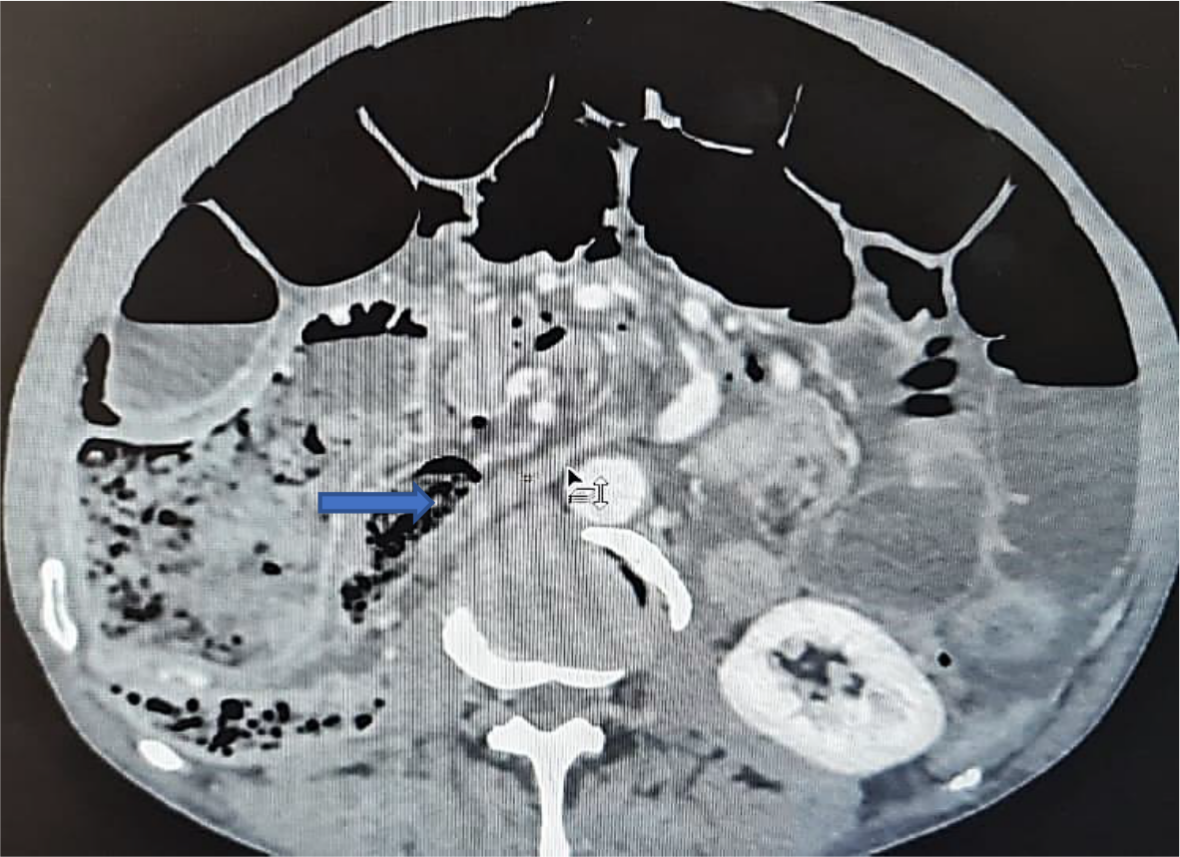
The inflammatory appendix.

An abdominal CT scan was performed, revealing distended small bowel loops upstreaming transitional levels like a ‘whirl sign’ ( Figure 2), a bubbly pattern across the length of the small bowel associated with parietal suffering signs ( Figure 3), abundant pneumoperitoneum ( Figure 4), and a pathological meso-celiac appendix ( Figure 5). The CT scan suggested a diagnosis of ileal volvulus due to the meso-celiac appendix.

**Figure 6.**
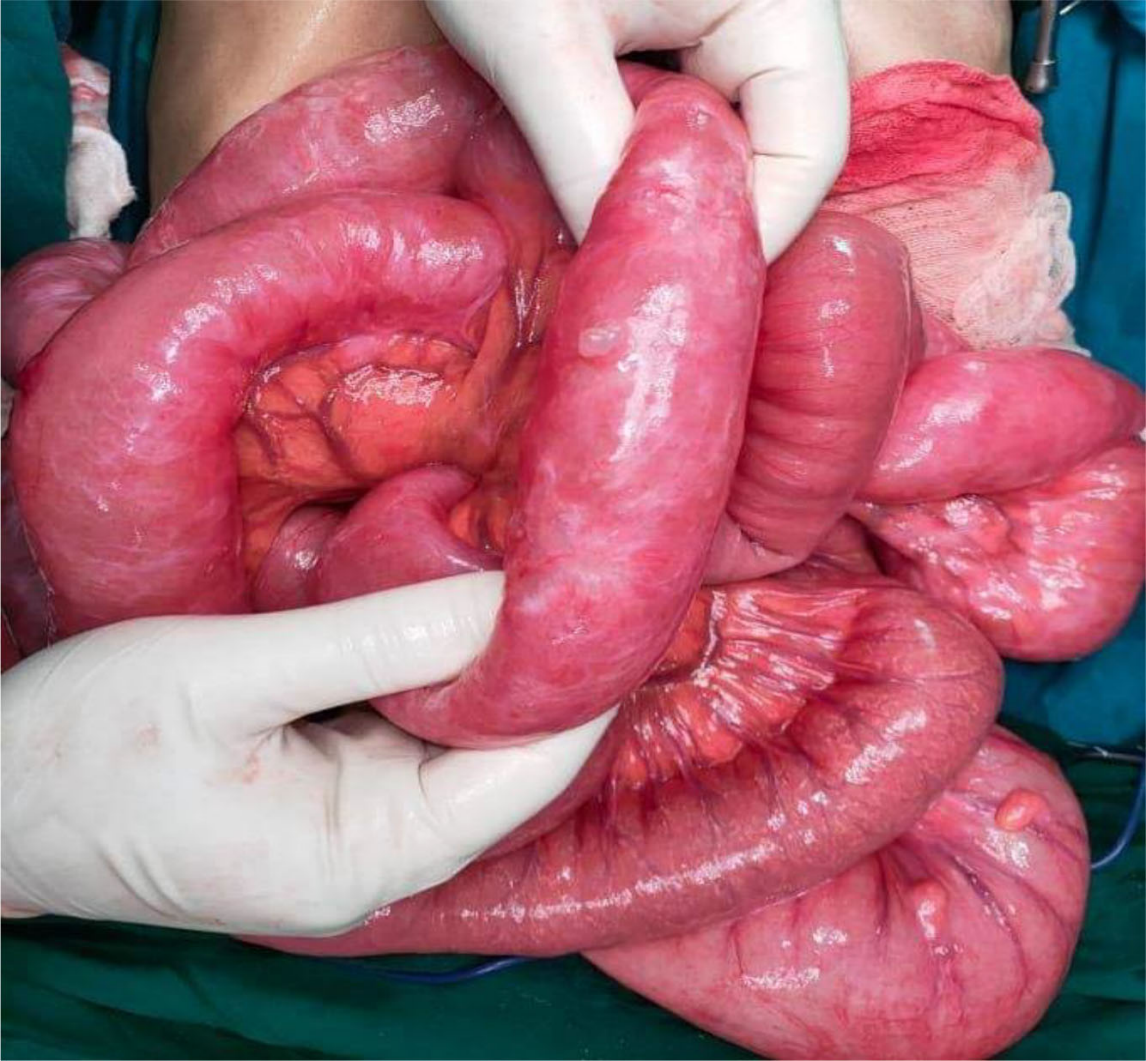
Ileal loop volvulus, involving a two-and-a-half clockwise turn around a long, pendulous small bowel mesentery.

**Figure 7.**
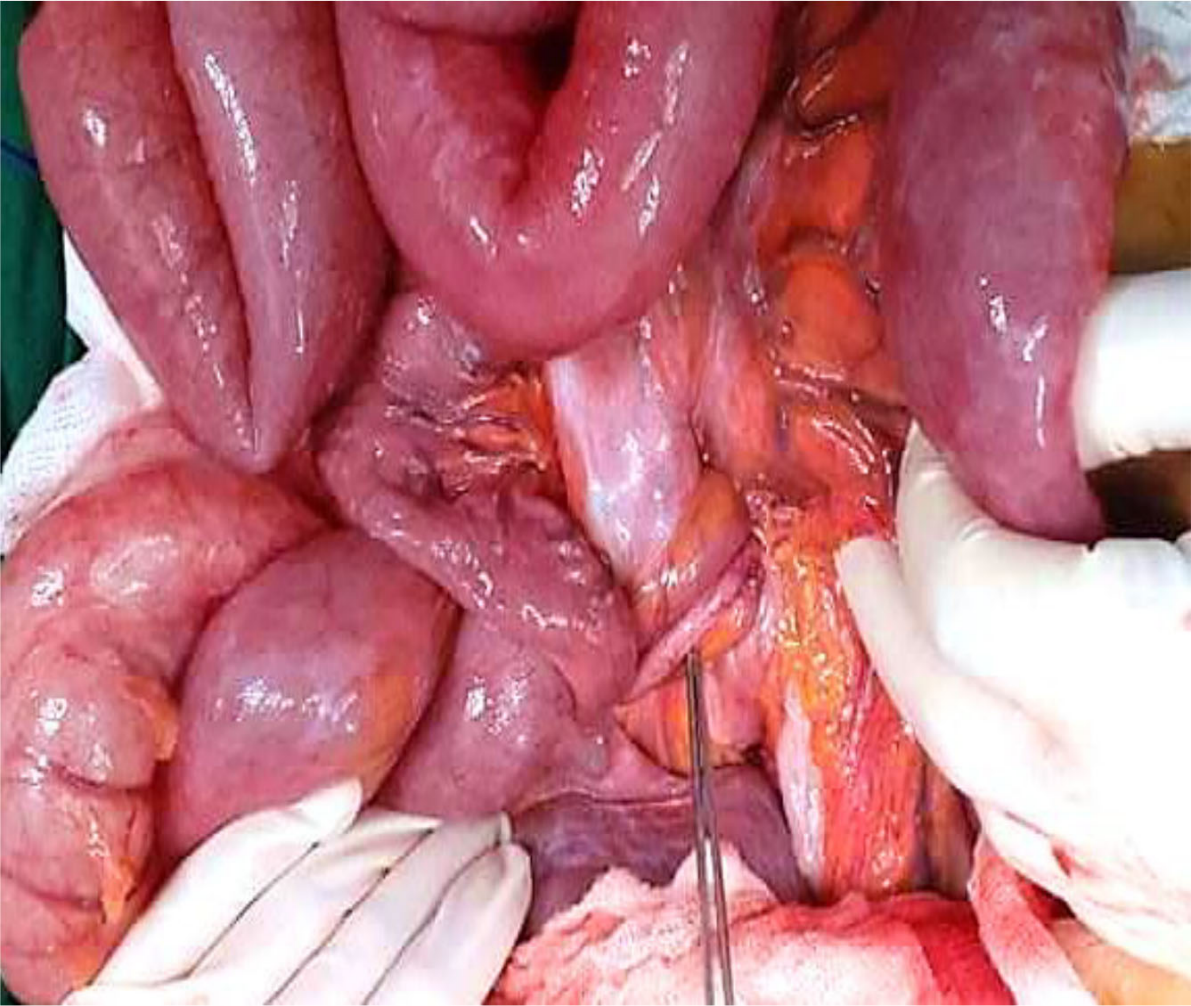
At the base of the volvulus, the inflammatory appendix wrapped around the last loop of the ileum.

**Figure 8.**
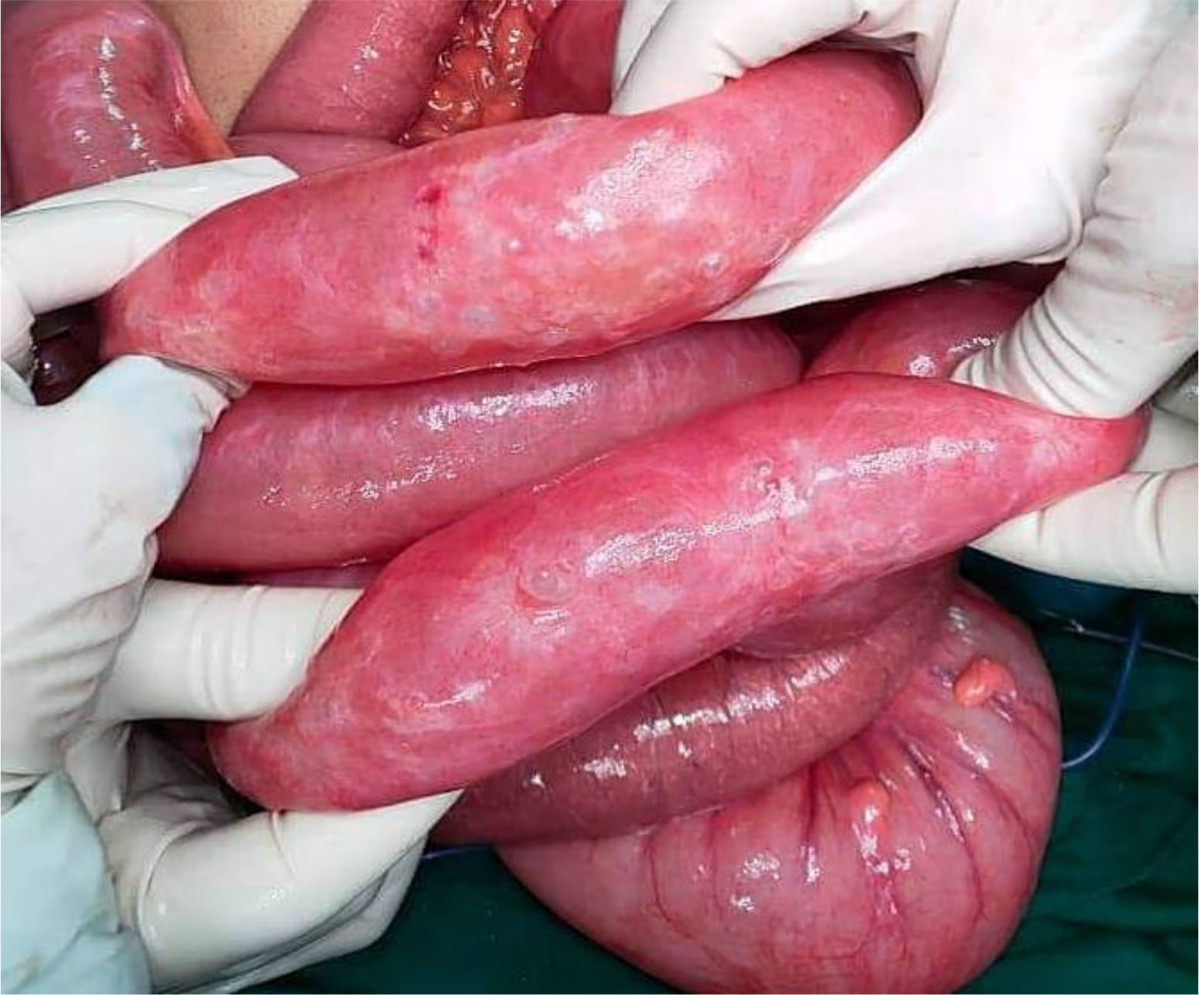
Segmental pneumatosis intestinalis involving the ileal loops.

We initially decided to perform laparoscopy. Intraoperatively, small bowel loops were much dilated not allowing intraperitoneal exploration. Gas-filled cystic lesions on small bowel serosa were identified. There was no evidence of perforation. We did choose to convert into midline incision for better and prudent exploration. A volvulus was found, involving a two-and-a-half clockwise turn around a long, pendulous small bowel mesentery, the strangled bowel was greatly congested ( Figure 6). At the base of the volvulus, an inflammatory appendix was wrapped around the last loop of the ileum ( Figure 7). Also, multiple gas-filled subserosal cysts, differently sized, on the wall of the ileum were encountered ( Figure 8). When the ileum was re-rotated, small bowel loops had preserved vitality. The entire colon was normal. Detorsion, retrograde draining, and appendectomy were performed because there were no signs of necrosis and the appendix was pathological.

Postoperatively, the patient completed a five-day course of intravenous metronidazole 500 mg three times a day. There were no postoperative complications. Anatomopathological examination revealed an inflammatory appendix without malignancy. A lower endoscopy was completed after surgery. It showed the presence of two polyps on the rectum and the transverse colon in low-grade dysplasia with no other lesions that were resected. There was no malignancy on the anatomopathological examination. The patient was monitored regularly, and the long-term post-operative course was uneventful.

## Discussion

PCI is an uncommon disease (0.03% of adults) ^6^ and its pathogenesis is still not clear. ^7^ According to its etiology, literature classifies this entity mainly as primary or secondary type. ^8^ There is also an idiopathic type which usually affects the left colon and is rarely reported in the literature. We found that thirteen cases of primary PCI have been described in the international literature ( Table 1). The secondary type frequently affects the small intestine and the right colon. ^9^ Its pathogenesis is multifactorial and can be explained by 3 theories: mucosal disruption, bacterial theory, and pulmonary disease. ^1, 7, 10, 11^ The mucosal disruption is due to the dissemination of bowel gas through a mucosal defect into lymphatic channels. ^1, 10^ Wu *et al.* ^12^ found that high altitude is a new theory explaining PCI’s pathogenesis. Highland areas induce passage of intraluminal gas into the submucosa damaging the mucosa. Mucosal damage can result from bowel occlusion, inflammatory process, and cytotoxic medical treatment. ^11^ The pulmonary cause is confirmed in patients with asthma and chronic bronchitis. In these cases, the rupture of alveoli causes the migration of air bubbles from interstitial spaces through the mediastinum and from the retroperitoneum to the blood vessel of the intestinal wall. ^1, 8, 10^ However, the bacterial theory is explained by entry of bacterial gas due to a defect on the bowel wall lymphoid tissue. ^8, 10^ This mechanism can justify the use of antibiotics. ^1^ Chemotherapy or hormonal therapy, and systemic sclerosis were also reported in the literature as a cause of PCI. ^1^ Finally, while keeping in mind these theories, their pathogenesis has not been yet fully clarified. ^1^

**Table 1.** Review of the literature (2008-2021) illustrating cases of idiopathic PCI, and PCI secondary to surgical etiologies.

Author	Age	Physical examination	Abdominal radiography	CT scan	Treatment	Etiology of PCI
Year	M/F					
**González *et al*.** ^20^ **2021**	66 Y	Normal	__	Intestinal pneumatosis	Conservative: surveillance	Idiopathic
	M					
**Moyon *et al*.** ^6^ **2020**	79 Y	Tachycardia Abdominal distension Mild pain on the lower abdomen without tenderness	--	The bubbly pattern across the length of the small bowel, Multiple cystic round shapes in the wall of the jejunum, and its mesentery, Large pneumoperitoneum	1/ *Conservative:* surveillance+ oxygen+ broad-spectrum antibiotics 2/Worsening pain *laparoscopy* : No evidence of perforation/gas-filled cystic lesions	Idiopathic
	M					
**Takahashi *et al*.** ^21^ **2019**	17 Y	Mobile mass in the right lower quadrant	__	Colocolic intussusception of the ascending colon with air in the bowel wall	*Endoscopy:* fine-needle aspiration	Idiopathic
	F					
**Suda *et al*.** ^22^ **2018**	80 Y	Slight abdominal distention	Dilatation and retention of gas in a segment of the small intestinal wall	Massive gas-filled cysts within the wall and mesentery of the small intestine No portal venous gas No intestinal ischemia	*Conservative:* antibiotics	Idiopathic
	M					
**Wang *et al*.** ^23^ **2018**	56 Y	Normal	Normal	--	*High-frequency electrosurgical resection* of the gas cysts + *ATB Bifidobacterium*	Idiopathic
	M					
**Romano-Munive *et al*.** ^24^ **2017**	54 Y	Normal	__	Pneumatosis cystoides intestinalis	*Conservative*	Idiopathic
	F					
**Furihata *et al*.** ^15^ **2016**	81 Y	Severe epigastric tenderness + distension No peritoneal signs	Massive free gas bilaterally in the subdiaphragmatic spaces	Massive free gas bilaterally under the diaphragm	*Conservative:* intravenous infusion of antibiotics, and nasogastric intubation	Secondary to chronic obstipation
	M					
**Fraga *et al*.** ^25^ **2016**	66 Y	Abdominal distention No signs of peritonitis	--	Gas in the abdominal wall, at the level of the transverse and rectosigmoid colon	*Conservative*	Idiopathic
	F					
**Tseng *et al*.** ^26^ **2014**	50 Y	Abdominal distension without tenderness	Gas in the bowel wall	Pneumatosis intestinalis of the right side of the colon	*Conservative*	Idiopathic
	F					
**Slesser *et al*.** ^5^ **2011**	74 Y	Abdominal distention Soft and non-tender	Bilateral free subdiaphragmatic air	Extensive pneumatosis intestinalis involving the small bowel with free intraperitoneal air secondary to malrotation of the proximal small bowel	*Laparotomy:* Extensive and bulky pneumatosis intestinalis extending from the duodenal–jejunal flexure to the terminal ileum	Idiopathic
	M					
**Nagata *et al*.** ^27^ **2010**	23 Y	Tenderness in the right iliac fossa	--	Intussusception of the ascending colon Ovoid radiolucencies with smooth margins	Intussusception easily resolved by *colonoscopy* *Laparoscopy-assisted* partial ascending colectomy was performed 1 month after his initial presentation	Idiopathic
	M					
**Arora *et al*.** ^4^ **2009**	27 Y	Signs of acute abdomen	Gas under the diaphragm	--	*Laparotomy:* Perforated duodenal ulcer + The terminal 180 cm of the small bowel to the ileocecal junction showed multiple thin-walled, tense, air-filled cysts on the serosal surface → Limited right colectomy and resection of the involved small bowel	Secondary to a surgical cause
	M					
**Liau *et al*.** ^3^ **2008**	76 Y	Signs of generalized peritonitis	Massive subdiaphragmatic air and multiple dilated loops of small bowel with well-demarcated wall	Pneumoperitoneum gas-filled cysts on the wall of small bowel loops	*Laparotomy:* extensive small bowel infarction due to volvulus of most of the small bowels, multiple gas-filled subserosal cysts on the wall of the ileum small bowel resection+ jejunoileal anastomosis	Secondary to a congenitally long mesentery
	M					

Besides, the disease’s location on the digestif tractus may be helpful to guide the etiology. So pyloric stenosis or gastric cancer can lead to a proximal pathology; however distant one might be due to mesenteric ischemia or diverticulitis. ^9^

PCI is a rare entity reported in the literature, but nowadays PCI reports’ number has been increasing because of the widespread use of CT scan and colonoscopy. ^9^ This pathology is more frequently asymptomatic. ^1^ Whereas in some cases, they may present with symptoms such as abdominal pain, constipation, distension, diarrhea, or bleeding. ^8, 9^ Incidentally PCI can induce surgical complications such as bowel obstruction, intestinal perforation, volvulus, intussusception, and bleeding, which require surgical intervention. ^13^

Intestinal obstruction can be a rare complication of PCI. This event depends on the size and number of the cysts which lead in certain cases to a reduction of the intestinal lumen, volvulus, perforation, and hemorrhage. ^7, 11^ In the literature, PCI associated with volvulus is much more uncommon. Besides this association, one of the highlights of our case is the long and hypermobile small bowel mesentery. Moreover, PCI is discussed to be a mechanical factor leading to irreversible volvulus, also it is disputed that volvulus contributes to ischemia which is an etiological factor leading to PCI. ^3, 14^

Imaging findings may be helpful to confirm PCI diagnosis, especially on CT scans. ^1, 6^ Computed tomography can show a grape cluster aspect within the wall of the intestine. ^1^ Three patterns of pneumatosis have been reported in the literature using CT scan imaging: bubble cystoid, a linear pattern, and a circular pattern. ^1^

Pneumoperitoneum can be explained by the rupture of the cyst on the wall intestine, without any evidence of peritoneal irritation or digestive perforation, like in our case. So that we should be wise to correlate clinical and radiographic findings, when free air is present below the diaphragm in chest X-ray. ^8, 9^ Pneumatosis intestinalis and portomesenteric venous gas (PVG) are generally debated independently in the literature. This association of radiological findings usually concludes to the presence of mesenteric infarction, but they may indicate occasionally nonischemic conditions. So that their presence should not be always regarded as signs of severity. ^13, 15, 16^ Moreover, according to literature, the rare findings of PCI and PVG can be present in asymptomatic patients without ominous signs, as described in the series of Sooby *et al*., ^17^ including 88 patients with PCI/PVG of which 19 with benign PCI, and of these 19, 6 patients had both PCI and PVG. These patients were put under surveillance, and they had no uneventful recovery.

The management of PCI is not well established, there are no standard therapeutic rules. ^10^ However, the mandatory in its management is to judge whenever it is benign or life-threatening. ^11^ So that it is established that if a CT scan shows intestinal infarction, urgent surgery is mandated. If no signs of intestinal damage is found, a conservative treatment is regarded to be ideal. ^13^ The common conservative procedure is to use metronidazole Antibiotics, which affects intestinal bacteria by the suppression of hydrogen production, and hyperbaric oxygen therapy. ^2, 6, 8, 10^ Nevertheless, a surgical procedure is indicated in complications such as peritoneal irritation or intestinal obstruction. ^2, 10^

The second particularity in our case is that the volvulus of the small bowel is due to acute appendicitis. This entity is explained by the wrapping of the appendix, due to its particular length, around the ileum occurring volvulus and strangulation. ^18, 19^ According to the literature, this mechanism resulted from adhesion of the inflamed appendix to the posterior peritoneum forming a turn of the spire of the ileum last loop resulting in volvulus. ^18, 19^ In summary, our case is exceedingly rare in the literature, featured by the ileal volvulus due to appendicitis.

## Conclusion

PCI is a rare disease whose diagnosis is offering a challenge for surgeons. This rare condition can often be associated with benign diseases or it can be proof of intestinal necrosis. Although surgery is mandatory in the complicated pattern, the treatment of asymptomatic forms is more likely conservative. Besides, both surgical and medical approaches can efficiently compete with these challenging diagnoses.

## Data availability

All data underlying the results are available as part of the article and no additional source data are required.

## Consent

Written informed consent was obtained from the patient regarding the publication of this case report.
