# Supplementary material for: Case Report: An unusual case of small bowel volvulus due to appendicitis associated with pneumatosis intestinalis: Review of the literature
Source: F1000Res. 2022 Nov 24;10:951. Originally published 2021 Sep 21. [Version 2] doi: 10.12688/f1000research.73042.2 (PMC9706145; doi:10.12688/f1000research.73042.2)
Supplement: Peer review history

# Peer review: Reviewer response for version 2

**DOI:** 10.5256/f1000research.139083.r156495

**Published:** 2022-11-28

**Authors:** Cristian Mesina

**Affiliations:** 1 Department of Surgery, Craiova University of Medicine and Pharmacy, Craiova, Romania

No comments

Are enough details provided of any physical examination and diagnostic tests, treatment given and outcomes?

Partly

Is the case presented with sufficient detail to be useful for other practitioners?

Yes

Is sufficient discussion included of the importance of the findings and their relevance to future understanding of disease processes, diagnosis or treatment?

Yes

Is the background of the case’s history and progression described in sufficient detail?

Yes

Reviewer Expertise:

surgery

I confirm that I have read this submission and believe that I have an appropriate level of expertise to confirm that it is of an acceptable scientific standard.

# Peer review: Reviewer response for version 1

**DOI:** 10.5256/f1000research.76663.r94999

**Published:** 2021-10-20

**Authors:** Cristian Mesina

**Affiliations:** 1 Department of Surgery, Craiova University of Medicine and Pharmacy, Craiova, Romania

1. During the clinical examination of the abdomen it would have been better to explain what was found on the percussion of the abdomen.

 2. Digital rectal examination was to be included in the clinical examination of the patient.

 3. Biological paraclinical examinations should have been more extensive to highlight whether or not there existed the presence of toxic-septic shock.

 4. The intraoperative exploration of the peritoneal cavity, the cause of pneumoperitoneum, should have been better explained.

Are enough details provided of any physical examination and diagnostic tests, treatment given and outcomes?

Partly

Is the case presented with sufficient detail to be useful for other practitioners?

Yes

Is sufficient discussion included of the importance of the findings and their relevance to future understanding of disease processes, diagnosis or treatment?

Yes

Is the background of the case’s history and progression described in sufficient detail?

Yes

Reviewer Expertise:

surgery

I confirm that I have read this submission and believe that I have an appropriate level of expertise to confirm that it is of an acceptable scientific standard, however I have significant reservations, as outlined above.

# Response

**Published:** 2021-12-07

**Authors:** meryam meryam

**Affiliations:** Habib Thameur Hospital, Tunisia

Dear Sir,

 I appreciate the time and effort that you and the reviewers have dedicated to providing your valuable feedback on my manuscript. I am grateful to the reviewers for their insightful comments on our paper. I have been able to incorporate changes to reflect most of the suggestions provided by the reviewers.

 Thank you, sincerely.

# Peer review: Reviewer response for version 1

**DOI:** 10.5256/f1000research.76663.r94998

**Published:** 2021-10-13

**Authors:** Jaques Waisberg

**Affiliations:** 1 Department of Surgery, Institute of Medical Assistance to State Civil Servants (IAMSPE), Sao Paulo, Brazil

General considerations:

 The clinical relevance of pneumatosis intestinalis varies widely from benign to potentially fatal conditions, depending on the underlying cause of pneumatosis intestinalis, which can lead to high mortality if surgery is delayed when necessary. Mesenteric ischemia, intestinal obstruction, and intestinal necrosis represent the fatal causes of pneumatosis intestinalis. It is challenging to distinguish between those patients who require surgery and those who can be observed or treated conservatively with adequate treatment of the underlying disease. The diagnosis of intestinal pneumatosis is made by computed tomography or plain abdominal radiography. Computed tomography is the most sensitive imaging modality. A strong indicator of the presence of mesenteric infarction or ischemia is the association of pneumatosis intestinalis with gas in the portal venous blood.

 Surgery is not indicated in asymptomatic patients with radiographic signs of pneumatosis intestinalis but no signs of bowel obstruction or free peritoneal air. Intestinal ischemia is the most likely cause, and a delay in surgery will worsen the patient’s condition. When non-surgical treatment is initially performed, the patient must be re-evaluated later. Surgery must be performed if there is no response to conservative treatment or if the patient’s condition worsens.

 This is an exciting manuscript about a rare case of pneumatosis intestinalis in an adult patient. The subject covered by the manuscript is required for clinical practice. *This manuscript has scientific value due to the rarity of the case. However, this case report needs some adjustments.*

 Specific considerations:

 Title – Appendix necrosis is identified as one of the contributing causes for the onset of pneumatosis intestinalis. As the authors themselves state in the text, appendicitis was the determining factor for small bowel volvulus. The title should include this information. The authors perform a literature review (Table 1 and 27 references), so the term “literature review” needs to be in the title.

 Abstract – The conclusion that “This case report emphasizes the importance of surgical procedures in the management of symptomatic pneumatosis intestinalis” needs to be contextualized according to the underlying disease, as this is precisely the dilemma that is faced with distinguishing the clinical or surgical approach in symptomatic cases of pneumatosis intestinalis.

 Discussion – The terms “primary” and “idiopathic” pneumatosis intestinalis are equivalent, so I suggest unifying them. A thorough explanation of the theories of the pathogenesis of intestinal pneumatosis is not necessary for the case description. I also recommend not using the term “benign PCI,” just “PCI”. Is there a “malignant PCI”? What were the reasons for six patients with “benign” PCI and PVG “not having” or “having” an uneventful recovery? Please remove the term “Antibiotics” after the word “metronidazole”.

 Figures – The legends of figures 1 to 5 are incomplete, as they must be self-explanatory (Exam name, disease name). Highlights in the legend must be marked with arrows on the figure itself. In figures with arrows, the pointed structure must be indicated in the legend. Figures 6 and 7 are unnecessary, as they do not add relevant information to the report.

Are enough details provided of any physical examination and diagnostic tests, treatment given and outcomes?

Yes

Is the case presented with sufficient detail to be useful for other practitioners?

No

Is sufficient discussion included of the importance of the findings and their relevance to future understanding of disease processes, diagnosis or treatment?

No

Is the background of the case’s history and progression described in sufficient detail?

Yes

Reviewer Expertise:

Gastrointestinal Surgery

I confirm that I have read this submission and believe that I have an appropriate level of expertise to confirm that it is of an acceptable scientific standard, however I have significant reservations, as outlined above.

# Response

**Published:** 2022-10-16

**Authors:** meryam meryam

**Affiliations:** Habib Thameur Hospital, Tunisia

thank you for your efforts i made all the corrections

# Peer review: Reviewer response for version 1

**DOI:** 10.5256/f1000research.76663.r95001

**Published:** 2021-10-12

**Authors:** Gennaro Martines

**Affiliations:** 1 Department of Emergency and Organ Transplantation, University "Aldo Moro" of Bari, Bari, Italy

'Pneumatosis intestinalis (PI), described by Du Vernoy in 1730, is a rare condition that refers to the presence of multiple gaseous cysts in the intestinal submucosa and subserosa with a reported incidence of 0.03%. It can be divided between a benign form and in a life-threatening condition requiring surgery.

 The radiographic incidence of PI has been reported to be up to 0.37% of patients who have abdominal computed tomography (CT) scans. PI can be divided into the primary/idiopathic type (15%), which refers to air pockets that imply to a chronic and benign idiopathic etiology, and the secondary type (85%), which refers to radiological findings of linear, microvesicular, or more circumferential appearing intramural gas caused by several predisposing factors. The peak age at onset is 45.3 ± 15.6 years with a male to female ratio of 2.4: 1.' ^1^

 This is an interesting paper which describes an unusual case of pneumatosis intestinalis. This situation is associated with many causes of bowel obstruction, and often requires a surgical approach in an emergency.

 The manuscript is well described but I want to ask why the authors perform a laparoscopy. In these situations there is a distension of the bowel which does can make this kind of approach risky. Can they explain this decision as a surgical approach?

 All the other parts are well described with adequate diagnostic steps.

Are enough details provided of any physical examination and diagnostic tests, treatment given and outcomes?

Yes

Is the case presented with sufficient detail to be useful for other practitioners?

Yes

Is sufficient discussion included of the importance of the findings and their relevance to future understanding of disease processes, diagnosis or treatment?

Yes

Is the background of the case’s history and progression described in sufficient detail?

Yes

Reviewer Expertise:

general surgery

I confirm that I have read this submission and believe that I have an appropriate level of expertise to confirm that it is of an acceptable scientific standard, however I have significant reservations, as outlined above.

## References

[1] : Pneumatosis Intestinalis and Hepatic Portal Venous Gas: Watch and Wait or Emergency Surgery? A Case Report and Literature Review. *Am J Case Rep* .2020;21: 10.12659/AJCR.923831 e923831 10.12659/AJCR.923831 32653891PMC7377522

# Response

**Published:** 2022-10-16

**Authors:** meryam meryam

**Affiliations:** Habib Thameur Hospital, Tunisia

the first laparoscopic pathway was indicated in the first place as an easy and minimally invasive exploratory approach.
